# A re-testing range is recommended for ^13^C- and ^14^C-urea breath tests for *Helicobacterpylori* infection in China

**DOI:** 10.1186/s13099-021-00435-3

**Published:** 2021-06-12

**Authors:** Xiangyu Wang, Shuzhen Zhang, Eng Guan Chua, Yongsheng He, Xiaofeng Li, Aijun Liu, Haiting Chen, Michael J. Wise, Barry J. Marshall, Dayong Sun, Xuehong Li, Chin Yen Tay

**Affiliations:** 1 Department of Gastroenterology, Health Science Center, Shenzhen Second People’s Hospital, The First Affiliated Hospital of Shenzhen University, Shenzhen, 518035 China; 2 Department of Clinical Laboratory, Kuichong People’s Hospital, Shenzhen, 518116 China; 3 The Marshall Centre for Infectious Diseases Research and Training, University of Western Australia, Perth, 6009 Australia; 4 Department of Gastroenterology, Kuichong People’s Hospital, Shenzhen, 518116 China; 5 Department of Nuclear Medicine, Shenzhen People’s Hospital, Shenzhen, 518001 China; 6 Department of Computer Science and Software Engineering, University of Western Australia, Perth, 6009 Australia

**Keywords:** *Helicobacter pylori*, Urea breath test, Diagnostic performance, Indeterminate range

## Abstract

**Background:**

The urea breath test (UBT) is widely used for diagnosing *Helicobacter pylori* infection. In the Shenzhen Kuichong People’s Hospital, some UBT findings were contradictory to the histology outcomes, therefore this study aimed to assess and compare the diagnostic performance of both ^13^C- and ^14^C-UBT assays.

**Methods:**

We recruited 484 *H. pylori*-treatment naïve patients, among which 217 and 267 were tested by the ^13^C-UBT or ^14^C-UBT, respectively. The cutoff value for *H. pylori* positivity based on manufacturer’s instruction was 4% delta over baseline (DOB) for the ^13^C-UBT, and 100 disintegrations per minute (DPM) for the ^14^C-UBT. Gastric biopsies of the antrum and corpus were obtained during endoscopy for histopathology.

**Results:**

In patients who were tested using the ^13^C-UBT kit, histopathology was positive in 136 out of 164 UBT-positive patients (82.9% concordance), and negative in 46 out of 53 UBT-negative cases (86.8% concordance). For the ^14^C-UBT-tested patients, histopathology was positive for *H. pylori* in 186 out of 220 UBT-positive patients (84.5% concordance), and negative in 41 out of 47 UBT-negative cases (87.2% concordance). While the ^13^C-UBT and ^14^C-UBT each had a high sensitivity level of 95.1% and 96.9%, respectively, their specificity was low, at 62.2% and 54.7%, respectively. By using new optimal cutoff values and including an indeterminate range (3–10.3% DOB for ^13^C-UBT and 87–237 DPM for ^14^C-UBT), the specificity values can be improved to 76.7% and 76.9% for the ^13^C- and ^14^C-UBT, respectively.

**Conclusions:**

The establishment of an indeterminate range is recommended to allow for repeated testing to confirm *H. pylori* infection, and thereby avoiding unnecessary antibiotic treatment.

*Trial registration*: Chinese Clinical Trial Registry, ChiCTR2000041570. Registered 29 December 2020- Retrospectively registered, http://www.chictr.org.cn/edit.aspx?pid=66416&htm=4

**Supplementary Information:**

The online version contains supplementary material available at 10.1186/s13099-021-00435-3.

## Introduction

*Helicobacter pylori* infection is common in China, with an overall estimated prevalence of 55.8% [1]. It is an important gastric pathogen that can lead to several gastroduodenal disorders including chronic gastritis, gastric atrophy and peptic ulcer disease, and less commonly, to gastric adenocarcinoma and mucosa associated lymphoid tissue (MALT) lymphoma [2, 3].

*Helicobacter pylori* is able to convert urea into carbon dioxide and ammonia via its urease enzyme, where the ammonia is used to neutralize the acid for its survival in the stomach [4]. Based on this feature of *H. pylori*, the urea breath test (UBT), a non-invasive *H. pylori* infection diagnostic method was developed. This requires a patient to swallow a capsule containing a dose of urea labeled with carbon-13 (^13^C) or carbon-14 (^14^C) isotope. If the patient is an *H. pylori* carrier, the labeled urea will be hydrolyzed by the bacterial urease enzyme within the stomach, resulting in the release of labeled carbon dioxide which is then absorbed into the bloodstream and expelled from the lungs in a few minutes. Hence the amount of labeled carbon dioxide within a patient’s breath sample can be measured to determine current *H. pylori* infection status [5, 6].

Due to its accuracy, simplicity and non-invasive nature, the UBT has been the preferred method of many medical professionals for testing *H. pylori* infection in their patients. Both the ^13^C-UBT and the ^14^C-UBT are widely used. The former utilizes the stable ^13^C isotope of carbon while the latter uses the radioactive ^14^C carbon isotope. Nevertheless, it is important to mention that both are naturally occurring isotopes and the radiation exposure from the ^14^C-UBT is even lower than that from background radiation [7]. In fact, the ^14^C-UBT has been approved by the Food and Drug Administration (FDA) of the United States for its usage in everyone, including children and pregnant women [8].

While UBT is useful in detecting *H. pylori* infection, we noticed that several UBT results were contradictory to the outcomes determined via histopathology examination, prompting us to reconsider the diagnostic accuracy of the commercial UBT kits used for screening *H. pylori* infection in our hospital. In this study, we recruited 484 individuals who underwent endoscopic examination at Shenzhen Kuichong People’s Hospital, among which 217 and 267 were tested for *H. pylori* infection using the ^13^C-UBT and ^14^C-UBT, respectively. By comparing the outcomes to that of histopathology examination of gastric biopsies, which is the gold standard for diagnosing *H. pylori* infection, we assessed the diagnostic performance of both UBT kits. Additionally, as these commercial kits available for use at our hospital provide only a cutoff value for *H. pylori* positivity, resulting in high positive rates, we therefore sought to introduce an “indeterminate zone”. Should a UBT value fall within the indeterminate range, we would like to recommend a repeat UBT or the use of another *H. pylori* diagnostic method to confirm the presence or absence of an infection.

## Material and methods

### Overview of entire study

**Fig. 1:**
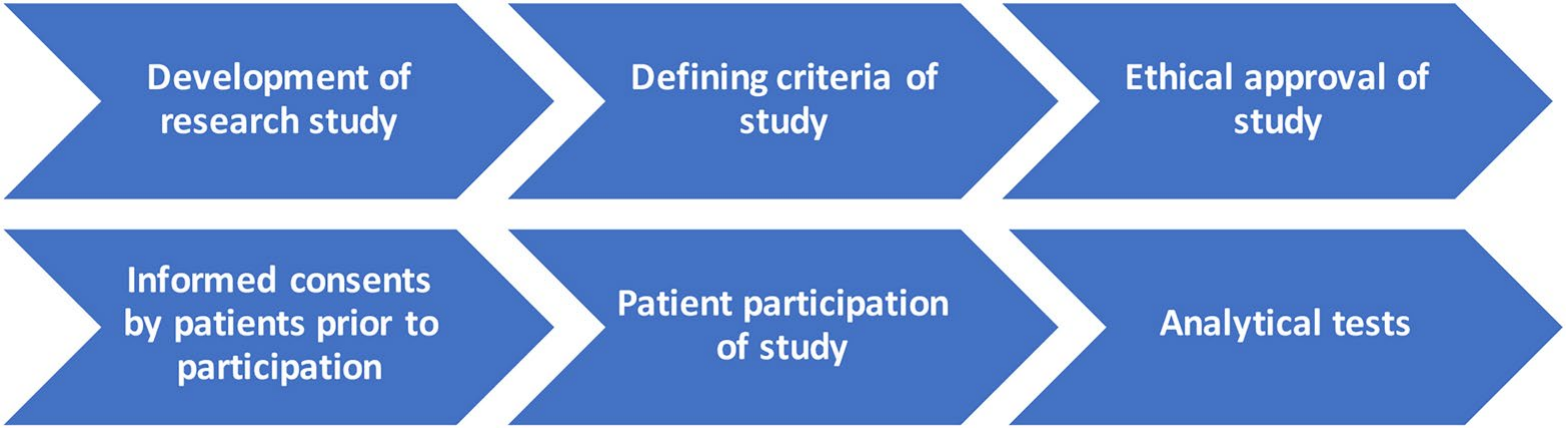
The overview of entire study

The schematic flow of experimental program was shown in Fig. 1.

#### Development of research study

During the clinical practice, we noticed that several UBT results were contradictory to the outcomes determined via histopathology examination, prompting us to reassess the diagnostic performance of both ^13^C-UBT and ^14^C-UBT commercial kits used for screening *H. pylori* infection in our hospital.

#### Defining the criteria of study

The following exclusion criteria on our study subjects were applied: (i) previous treatment of *H. pylori* infection, (ii) received proton pump inhibitor, H_2_ receptor antagonist, expectorant, or antibiotic treatment within the last four weeks prior to endoscopy, (iii) history of gastric surgery, (iv) diagnosed with gastrointestinal cancer, (v) had severe heart, lung, liver, kidney or blood system disorder, (vi) aged below 18 or above 70 years old and (vii) pregnancy.

#### Ethics approval

This study was approved by the research ethics committee of Shenzhen Kuichong People’s Hospital (Reference no. 201609) and registered at www.chictr.org.cn (Reference no. ChiCTR2000041570). Each patient was given a detailed introduction to the purpose and process of the research by a gastroenterologist. Written and informed consents were obtained from all patients prior to their participation in the study.

#### Study population

From January 2017 to November 2018, following the application of the exclusion criteria, 484 patients (18–70 years of age) who visited Shenzhen Kuichong People’s Hospital (26 Kuixin N Road, Dapeng New District, Shenzhen City, China) for endoscopic check-up agreed to participate in this study.

Prior to the endoscopy session, the patients received either a ^13^C-UBT or a ^14^C-UBT at the discretion of a gastroenterologist via the alternating assignment method. The general health information of patients was collected by clinical nurses. During endoscopy, two gastric biopsy specimens (one each from antrum and corpus) were collected for histopathology examination. The histology examination was performed by Da’an Clinical Laboratory, a third-party pathology service provider. The UBTs were conducted by clinical technicians who had no knowledge of this study.

### Sample size determination

The samples size required for this study was estimated based on a 95% confidence interval using the following formula [9]:

Sample size (n1) based on sensitivity $= \frac{{Z_{{1 - \frac{a}{2}}}^{2} {\text{~Sens}}\left( {1 - {\text{Sens}}} \right)}}{{d^{2} Prev}}$

Sample size (n2) based on specificity $= \frac{{Z_{{1 - \frac{a}{2}}}^{2} {\text{~Spec}}\left( {1 - {\text{Spec}}} \right)}}{{d^{2} \left( {1 - Prev} \right)}}$ where Z, the normal distribution value, was set to 1.96 as corresponding with the 95% confidence interval, and *d*, the maximum acceptable width of the 95% confidence interval, was set to 10%. Based on a previous study, the UBT was shown to achieve a sensitivity value of 96% and a specificity value of 93% [10]. The prevalence rate (Prev) of *H. pylori* in Shenzhen, China was 35.85% [11]. Based on the criteria above, this study required at least 41 *H. pylori*-positive patients (n1) and 39 *H. pylori*-negative patients (n2), yielding a minimum total sample size of 80 participants for each UBT test.

### Urea breath test

#### ^*13*^*C-UBT*

The ^13^C-UBT (Beijing Boran Pharmaceutical Co. Ltd., China) was performed according to the manufacturer’s instructions. Briefly, an initial baseline breath sample was collected from each patient after fasting for at least four hours prior to ingesting a capsule containing 50 mg ^13^C isotope labeled urea with 80–100 mL of water. After 30 min of sitting, exhaled breath was again collected. The ^13^CO_2_ content within the initial and 30-min expiratory air bags were analyzed using an HG-IRIS13C infrared spectrometer (Beijing Richen-Force Science & Technology Co., China). Following 30 min of administration, a delta over baseline (DOB) value of 4% or above was regarded as a positive indicator of *H. pylori* infection.

#### ^14^C-UBT

The ^14^C-UBT (Zhonghe Headway Bio-Sci & Tech Co. Ltd., China) was performed according to the manufacturer’s instruction. Briefly, patients who fasted for at least four hours were requested to ingest a gelatin capsule containing 0.75 µCi of ^14^C isotope with 20 mL of water. After 25 min, each patient was then asked to exhale continuously into a bottle until the purple-colored CO_2_ capturing liquid within turned colorless. The scintillation fluid was subsequently added, and the homogenized solution was measured for ^14^CO_2_ quantity. A reading with more than 100 disintegrations per minute (DPM) was classified as *H. pylori* positive.

### Histopathology

Two gastric biopsy specimens (one each from antrum and corpus) were sent to Da’an Clinical Laboratory (Guangzhou, China) for histopathology examination. The histopathologists were unaware of the UBT results. The presence of *Helicobacter-*like organism was confirmed with routine hematoxylin and eosin (HE) staining. Giemsa staining was further performed if HE could not confirm the presence of *H. pylori* clearly, and in those patients who had chronic active gastritis, but no *H. pylori* found in HE stains.

### Statistical analysis

The sensitivity and specificity values of each UBT method were reported according to manufacturer’s recommended cut-off value. To evaluate the diagnostic capacity of each UBT method, the receiver operating characteristic (ROC) curve was generated by plotting the true-positive rate on the y axis against the true-negative rate on the x axis [12]. Our ROC analysis was performed using the R package pROC (version 1.16.2) [13]. The area under the curve (AUC) was calculated to quantify the overall accuracy of each UBT method to diagnose *H. pylori* infection outcomes. The optimal cutoff UBT value that generates the highest true positive rate together with the lowest false positive rate, was determined by using maximum Youden index method, where Youden index = sensitivity + specificity − 1 [14]. For the comparison of categorical variables, the Fisher’s exact test was used. The level of statistical significance was considered at *p* < 0.05.

## Results

### Diagnostic performance of ^13^C-UBT and ^14^C-UBT with the manufacturer's recommended cutoff for UBT

The UBT readings, histological findings of *H. pylori* in gastric biopsies and patient demographics including age and sex are available in Additional file 1: Table S1. Among the 484 patients recruited in this study, 217 and 267 were tested using the ^13^C- and ^14^C-UBT kits, respectively. The numbers of *H. pylori*-positive and *-*negative patients were 164 (75.6%, 164/217) and 53 (24.4%, 53/217), using the ^13^C-UBT, and 220 (82.4%, 220/267) and 47 (17.6%, 47/267), as indicated by the ^14^C-UBT.

We next assessed the diagnostic performance of both UBT assays (Table 1). While the ^13^C-UBT and ^14^C-UBT each had a high sensitivity of 95.1% (CI 89.8%–97.8%) and 96.9% (CI 93.0%–98.7%), respectively, their specificity was unsatisfactory, at 62.2% (CI 50.1%–73.0%) and 54.7% (CI 42.8%–66.1%), respectively.

**Table 1:** Diagnostic performance of the ^13^C-UBT and ^14^C-UBT

Method	Histology			Sens (%)	Spec (%)	Acc (%)	FPR (%)	FNR (%)
		+	−	95%CI	95%CI	95%CI	95%CI	95%CI
^13^C-UBT	+	136	28	95.1	62.2	83.9	37.8	4.9
	−	7	46	89.8–97.8	50.1–73.0	78.4–88.2	27.0–49.9	2.2–10.2
^14^C-UBT	+	186	34	96.9	54.7	85	45.3	3.1
	−	6	41	93.0–98.7	42.8–66.1	80.2–88.8	33.9–57.2	1.3–7.0

*UBT* urea breath test, *Sens* sensitivity, *Spec* specificit, *Acc* accurac, *FPR* false positive rat, *FNR* false negative rat, *CI* confidence interval

We also compared the discordance of *H. pylori* infection status as determined by each UBT assay to histopathology in three different age groups (Table 2). Interestingly, in patients aged 18–30 years, there was a significantly higher discordance between the ^13^C-UBT and histopathology outcomes as compared to the ^14^C-UBT counterparts (29.4% versus 8.3%, *P* = 0.032). On the other hand, in patients aged above 50 years, the discordance was significantly greater in the ^14^C-UBT group than those who were tested by the ^13^C-UBT (28.4% versus 12.2%, *P* = 0.045). No significant difference was observed in the 31–50 years patient group.

**Table 2:** Discordance between UBT and histopathology findings among different patient age groups

Age (years)	^13^C-UBT			^14^C-UBT			*P*
	# FP	# FN	Discordance [% (n/N)]	# FP	# FN	Discordance [% (n/N)]	
18–30	10	0	29.4 (10/34)	3	0	8.3 (3/36)	0.032
31–50	15	4	14.2 (19/134)	12	4	10.2 (16/157)	0.367
> 50	3	3	12.2 (6/49)	19	2	28.4 (21/74)	0.045

The distribution of discordant findings between both UBT assays in each age group was tested using the Fisher’s exact test, with a *P* value less than 0.05 considered as statistically significant

*FP* false positive; *FN* false negative

### ROC analysis and development of optimal cutoff values

**Fig. 2:**
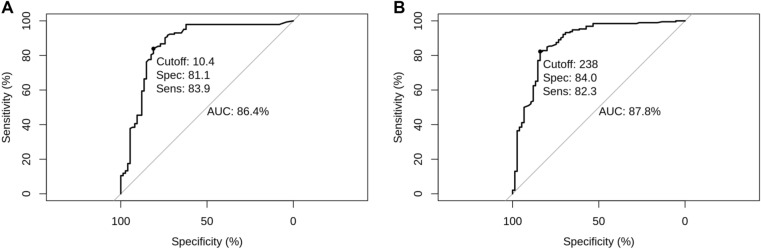
ROC curve of UBT for the diagnosis of *H. pylori* infection. **A** ROC curve of ^13^C-UBT. **B** ROC curve of ^14^C-UBT

Using a ROC analysis, the optimal cutoff value as a positive indicator for *H. pylori* infection was 10.4% DOB for ^13^C-UBT, and 238 DPM for ^14^C-UBT (Fig. 2). With these cutoffs, the AUCs for ^13^C- and ^14^C-UBT were 86.4% and 87.8%, respectively. While the increase in cutoff value had greatly reduced the number of false positives in each UBT assay, improving the specificity from 62.2% (CI 50.1%–73.0%) to 81.1% (CI 70.0%–88.9%) for ^13^C-UBT, and from 54.7% (CI 42.8%–66.1%) to 84% (CI 73.3%–91.1%) for ^14^C-UBT, the sensitivities decreased to 83.9% (CI 76.6%–89.3%) and 82.3% (CI 76.0%–87.3%) (Table 3).

**Table 3:** Diagnostic performance of the ^13^C-UBT and ^14^C-UBT with optimal cutoff values for *H. pylori* positivity

Method	Histology			Sens (%)	Spec (%)	Acc (%)	FPR (%)	FNR (%)
		+	–	95%CI	95%CI	95%CI	95%CI	95%CI
^13^C-UBT	+	120	14	83.9	81.1	82.9	18.9	16.1
	–	23	60	76.6–89.3	70.0–88.9	77.4–87.4	11.1–30.0	10.7–23.4
^14^C-UBT	+	158	12	82.3	84	82.8	16	17.7
	–	34	63	76.0–87.3	73.3–91.1	77.8–86.9	8.9–26.7	12.7–24.0

Based on the maximum Youden index method, the optimal cutoff values for *H. pylori* positivity were 10.4% DOB and 238 DPM for the ^13^C-UBT and ^14^C-UBT, respectively

*UBT* urea breath test, *Sens* sensitivity, *Spec* specificity, *Acc* accuracy, *FPR* false positive rate, *FNR* false negative rate, *CI* confidence interval

**Fig. 3:**
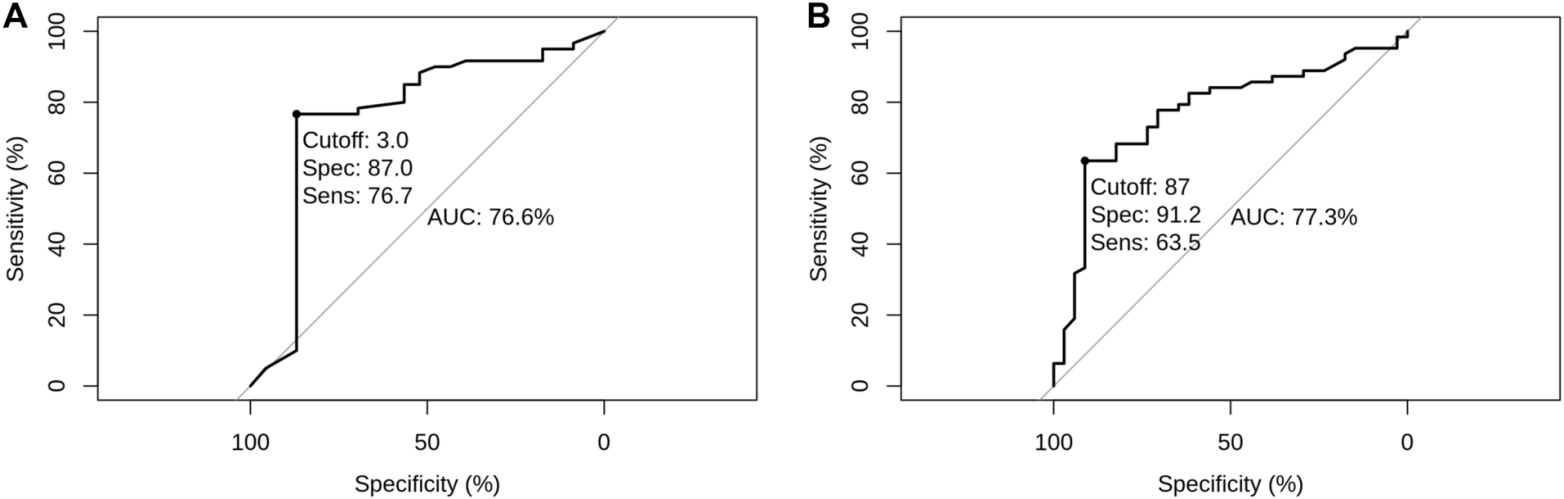
ROC curve of UBT for the diagnosis of H. pylori infection with only UBT readings below the optimal positive cutoff value. **A** ROC curve of ^13^C-UBT. **B** ROC curve of ^14^C-UBT

To improve the sensitivity of each assay, we thought that it was necessary to establish an additional cutoff value as a negative indicator of *H. pylori* infection, following which the values situated between the upper and lower cutoffs would be classified as indeterminate results and therefore require repeated testing. Again, ROC analysis was performed on each UBT assay and this time, only with values below the optimal cutoff as previously determined. As shown in Fig. 3, the new cutoff value was 3% DOB for ^13^C-UBT, and 87 DPM for ^14^C-UBT, which in turn implies that UBT values less than 3% DOB or 87 DPM were very likely to be *H. pylori* negative. Taking both upper and lower cutoff values for each assay into consideration, we recommend that for ^13^C-UBT readings of 3% to 10.3% and ^14^C-UBT readings of 87 to 237, the *H. pylori* infection status should be considered indeterminate and would therefore require a repeated testing.

More importantly, with the introduction of an indeterminate zone, in which its (indeterminate) readings have been excluded from performance assessment and subjected to other test methods, the sensitivity and specificity can be improved to 96% (CI 90.4%–98.5%) and 76.7% (CI 63.7%–86.2%) for ^13^C-UBT, and 98.1% (CI 94.2%–99.5%) and 76.9% (CI 62.8%–87.0%) for ^14^C-UBT (Table 4). Further, the diagnostic accuracy of the ^13^C- and ^14^C-UBT in this population can be boosted from initially 83.9% (CI 78.4%–88.2%) and 85% (CI 80.2%–88.8%), to 89.7% (CI 84.5%–93.4%) and 93% (CI 88.6%–95.8%), respectively.

**Table 4:** Diagnostic performance of the ^13^C-UBT and ^14^C-UBT with optimal *H. pylori*-positive and -negative cutoff values, and the inclusion of an indeterminate range

Method	Histology			Sens (%) 95%CI	Spec (%) 95%CI	Acc (%) 95%CI	FPR (%) 95%CI	FNR (%) 95%CI
		+	–					
^13^C-UBT	+ (≥ 10.4%)	120	14	96 90.4–98.5	76.7 63.7–86.2	89.7 84.5–93.4	23.3 13.8–36.3	4 1.5–9.6
	− (≤ 2.9%)	5	46					
	IND (3–10.3%)	18	14					
^14^C-UBT	+ (≥ 238 DPM)	158	12	98.1 94.2–99.5	76.9 62.8–87.0	93 88.6–95.8	23.1 13.0–37.2	1.9 0.5–5.8
	− (≤ 86 DPM)	3	40					
	IND (87–237 DPM)	31	23					

*UBT* urea breath test, *IND* indeterminate, *Sens* sensitivity, *Spec* specificity, *Acc* accuracy, *FPR* false positive rate, *FNR* false negative rate, *CI* confidence interval

## Discussion

The urea breath test is widely accepted as an accurate non-invasive method for diagnosing *H. pylori* infection. In the present study, we assessed the diagnostic performance of the ^13^C- and ^14^C-UBT commercial kits used in our hospital by comparing each UBT outcome against that of histological examination, which was considered the “gold standard” reference method for determination of *H. pylori* infection. Importantly, based on histology, the ^14^C-UBT was significantly more accurate than the ^13^C-UBT in determining *H. pylori* infection status in patients aged 18–30 years, whereas, in patients older than 50 years of age, the ^13^C-UBT method was more accurate than the ^14^C-UBT.

The ^13^C-UBT measures the ratio of labeled CO_2_ to human respiratory CO_2_ in the breath. Hence its outcome can be affected by one’s gender, age, urea hydrolysis rate and CO_2_ production rate [15]. Therefore, in the event where there were many false-positive ^13^C-UBT results among the young patients, it is possible that these individuals have a relatively low basal CO_2_ production rate and/or a high urea hydrolysis rate, releasing breath with a proportionally higher quantity of labeled CO_2_ and thus, generating a false-positive DOB value. In the situation where ^14^C-UBT generated substantially more false-positive results than ^13^C-UBT in older patients, some of these patients might have hypochlorhydria, a condition where there is a low-level production of gastric acid and which is commonly associated with aging, leading to the growth of urease-producing non-*H. pylori* bacteria originating either from the oral cavity or the intestine and thus, a UBT-positive outcome [16–18].

Also, it is also possible that the four-hour fasting time (prior to ingesting a capsule containing 50 mg ^13^C isotope-labeled urea) of this current study is insufficient to empty the stomach in some of these individuals. This situation is most likely to generate a less-acidic gastric environment, which would be rather permissive for the growth of other bacteria with urease activity that could eventually induce a false-positive UBT reaction. Therefore, a longer fasting period, potentially overnight when possible, should be considered the preferred option before testing. Additionally, attention to detail when performing the tests could improve the accuracy. As an example, cleaning teeth and mouth immediately prior to the test might decrease gastric contamination from swallowed oropharyngeal (urease positive) bacteria. Ensuring the patient was sitting quietly prior to the test would lower the amount of endogenous CO_2_ resulting in a slightly higher breath enrichment of the isotope.

Depending on populations and the doses of ^13^C-urea or ^14^C-urea, no one-size-fits-all UBT cutoff value can be used to define whether an individual is *H. pylori*-positive or -negative [19–22]. In our study, to overcome the low specificity of each UBT kit, two optimal cutoff points, indicating UBT-positive and -negative, respectively, along with an indeterminate zone to address UBT readings that are inconclusive, were established. The intermediate zone, defined as ranging from 3% to 10.3% DOB for ^13^C-UBT, and from 87–237 DPM for ^14^C-UBT, contained at least half of the false-positive test results in this study. By using new optimal cutoff values and including an indeterminate range, the false positive rates can be greatly reduced. More importantly, we suggest that for any patient who had an indeterminate UBT result, a repeated UBT or other diagnostic test such as stool antigen or serum antibody test should be performed to confirm *H. pylori* infection, avoiding misdiagnosis and unnecessary antibiotic treatment.

## Limitations of study

We concede that there are limitations in this study which was performed in a busy clinical setting rather than in a formal research environment. Despite with the new cutoffs and the establishment of an indeterminate range, the specificity of each UBT kit was only improved to approximately 77%, which is still considerably lower than reported (10). The lower specificity of each UBT kit used in this study was probably due to the misdiagnosis of *H. pylori* infection when histology alone was used as the reference method, as its accuracy depends on the skills of the operator, the size and number of biopsies taken and whether or not the biopsy site contained *H. pylori* or missed it by chance.

To address this issue, a larger cohort study with a more even distribution of different age categories, as well as the ability to test every study participant using both ^13^C- and ^14^C-UBT, and the concordant use of two or three methods as references should be further performed. For example, rapid urease test, bacterial culture, histology and even PCR, would create a truer “gold standard” and allow for better comparison of the diagnostic accuracy of both tests and the validation of our suggested indeterminate zone. At the same time, observation of how the tests were administered in a busy clinical setting could increase the value of the test even further.

## Conclusions

Via comparing the UBT outcomes to that of histopathology examination, we demonstrated that both ^13^C- and ^14^C-UBT kits used in this study have high sensitivity but low specificity. Based on ROC analysis and the maximum Youden index method, new optimal cutoff values were identified and used to establish an indeterminate range (3–10.3% DOB for ^13^C-UBT and 87–237 DPM for ^14^C-UBT), improving the specificity from 62.2% to 76.7% and 54.7% to 76.9% for the ^13^C- and ^14^C-UBT, respectively. We strongly suggest that for any patient who had an indeterminate UBT result, a repeated UBT or other diagnostic test should be performed to confirm *H. pylori* infection, avoiding misdiagnosis and unnecessary antibiotic treatment. For future studies, a larger cohort study with two or three methods as references should be further performed to validate our suggested indeterminate zone.

## Supplementary Information

**Additional file 1: Table S1.** Patient demographics and the results for 13C-UBT, 14C-UBT, rapid urease testing, histology examination and bacterial culturing test.

## Data Availability

All data generated or analysed during this study are included in this published article (and its Additional files).
